# Unexplored diversity and strain-level structure of the skin microbiome associated with psoriasis

**DOI:** 10.1038/s41522-017-0022-5

**Published:** 2017-06-22

**Authors:** Adrian Tett, Edoardo Pasolli, Stefania Farina, Duy Tin Truong, Francesco Asnicar, Moreno Zolfo, Francesco Beghini, Federica Armanini, Olivier Jousson, Veronica De Sanctis, Roberto Bertorelli, Giampiero Girolomoni, Mario Cristofolini, Nicola Segata

**Affiliations:** 1 Centre for Integrative Biology, University of Trento, Trento, Italy; 2 Istituto G.B. Mattei, Comano, Italy; 3 NGS Facility, Laboratory of Biomolecular Sequence and Structure Analysis for Health, Centre for Integrative Biology, University of Trento, Trento, Italy; 4 Department of Medicine, Section of Dermatology, University of Verona, Verona, Italy

## Abstract

Psoriasis is an immune-mediated inflammatory skin disease that has been associated with cutaneous microbial dysbiosis by culture-dependent investigations and rRNA community profiling. We applied, for the first time, high-resolution shotgun metagenomics to characterise the microbiome of psoriatic and unaffected skin from 28 individuals. We demonstrate psoriatic ear sites have a decreased diversity and psoriasis is associated with an increase in *Staphylococcus*, but overall the microbiomes of psoriatic and unaffected sites display few discriminative features at the species level. Finer strain-level analysis reveals strain heterogeneity colonisation and functional variability providing the intriguing hypothesis of psoriatic niche-specific strain adaptation or selection. Furthermore, we accessed the poorly characterised, but abundant, clades with limited sequence information in public databases, including uncharacterised *Malassezia* spp. These results highlight the skins hidden diversity and suggests strain-level variations could be key determinants of the psoriatic microbiome. This illustrates the need for high-resolution analyses, particularly when identifying therapeutic targets. This work provides a baseline for microbiome studies in relation to the pathogenesis of psoriasis.

## Psoriasis: investigating microbial diversity

Analysing microbial populations on the skin provides an insight into the diversity of microbes associated with psoriasis. Nicola Segata and colleagues at the University of Trento, Italy, used genetic analysis to compare the microbial populations on regions of skin affected and unaffected by psoriasis. *Staphylococcus* bacteria were more prevalent in psoriasis, but there was little clearly defined difference in microbial species on psoriasis-affected and unaffected skin. There was, however, decreased microbial diversity on psoriatic ear sites. Deeper strain-level computational analysis suggested that psoriasis could offer niche locations for colonisation by specific strains of staphylococci and propionibacteria. The results highlight the diversity of microbial populations on the skin, and the need for larger cohorts to build on the baseline data now established. Further studies might help identify targets for treating skin bacteria associated with psoriasis.

## Introduction

The human skin is an interface between the host and the environment, providing bidirectional communication and effective protection from infectious microorganisms and toxic substances.^1^ It is a complex ecosystem whereby the resident microbiome, physiochemical conditions, and host biology interact at the system level.^2^ The skin is inhabited by diverse communities of microorganisms,^3–7^ the vast majority of which are benign commensals living in harmony with the host.^3^ They provide protection by directly restricting or inhibiting harmful microorganisms and there is increasing evidence that they also play a role in educating or enhancing the adaptive and innate immune responses of the host when encountering harmful invaders.^8–12^ As such, the skin microbiome is an important aspect of skin health that remains largely unexplored, especially in relation to skin diseases.

Psoriasis is a common immune-mediated inflammatory disease of the skin affecting around 2–3% of the world’s population.^13^ Five types of psoriasis have been described with chronic plaque psoriasis (psoriasis vulgaris) by far the most common form responsible for approximately 90% of cases.^13^ Most of the genetic loci predisposing an individual to psoriasis have been identified by genome-wide linkage analysis and are involved with both acquired and innate immunity,^13, 14^ and it has been demonstrated that an aberrant immune response to antimicrobial peptide LL-37 is likely involved in psoriasis immunopathogenesis.^15^ It is becoming increasingly evident that psoriasis has a microbial component and that infections may cause disease exacerbation.^13^ Moreover, keratinocytes, which are directly exposed to and sense the skin microbiome, trigger innate and adaptive immune responses in psoriasis.^16^ However, current knowledge of the psoriasis-associated microbial community has been obtained until relatively recently by conventional culture-dependent studies. These studies have suggested several microorganisms to be associated with disease exacerbation, including *Staphylococcus aureus*,^17^ *Streptococcus pyogenes*,^18^ and fungi such as *Malassezia*.^19^ However, such pre-genomic approaches are restricted to the cultivable proportion of the microbiome, which is limited to only a fraction of microbial species, implying that a proportion of the microbiome was left unexplored. Culture-independent studies based on ribosomal marker genes (such as 16S and 18S rRNA genes) have more recently revolutionised our understanding of the resident microbiome,^20^ by enabling the profiling of the microbial communities at the system level without introducing heavy biases towards cultivable microbes. A few studies have reported overall community changes with respect to psoriasis, but no single biomarker indicative of disease has been found.^21–25^ These studies have been insightful in understanding the taxonomic differences associated with psoriasis and provide a platform on which to build. However, rRNA-based studies are limited in their taxonomic resolution, suffer from PCR biases, and offer no direct assessment of the genomic structure and functional potential of the community.

New generation community-wide, whole-genome shotgun metagenomics offers the potential to overcome the limitations of PCR-based surveys.^26^ Shotgun metagenomics has proved effective in characterising the gut microbiome in complex diseases such as type-2 diabetes,^27^ inflammatory bowel disease^28^ and obesity,^29^ but applying it to the skin microbiome is more challenging because of lower starting biomass and a large fraction of contaminant human DNA. Only a few shotgun metagenomic studies have surveyed the microbiota associated with the skin, as part of the Human Microbiome Project,^6^ The Home Microbiome Project,^30^ Oh et al.^7^ and recently in association with atopic dermatitis.^31^ Additionally we have also recently validated our approach on healthy skin regions,^32^ including the elbow skin area that is highly relevant in psoriasis and has never been targeted before. What is evident is that the healthy skin is a dynamic environment populated by taxonomically and functionally diverse microorganisms. This microbial diversity is driven by niche-specific colonisation of the skins microenvironments and underpinned by a strong subject individuality. In particular, Oh et al.^7^ surveyed with species-level and subspecies-level resolution the microbiome structure associated with healthy skin, uncovering hidden diversity. Moreover, with a large number of metagenomic reads unable to be mapped to publically available reference genomes, a considerable proportion of the skin metagenome remains uncharacterised, the so-called “microbial dark matter”.^7, 33^ While shotgun metagenomic studies are fundamental to study the skin in relation to disease, to date, they have mainly focussed on the skin of healthy individuals.

In this study, we provide the first shotgun metagenomic assessment of the skin microbiome in relation to the skin disease, specifically plaque psoriasis. We focused on a study design accounting for inter-subject variability by sampling diseased and unaffected skin from contralateral sites of each patient. Focussing on two common sites of psoriatic manifestation (ear and elbow), we apply species and finer strain-level resolution approaches, as well as exploration of the skin’s “dark matter” to unravel the microbial signatures associated with psoriasis.

## Results and discussion

### Sampling and sequencing the psoriasis-associated skin microbiome

To investigate the skin microbiome in relation to psoriasis, 28 patients clinically diagnosed with plaque psoriasis were recruited from the Dermatology Department of the Santa Chiara Hospital in Trento (Italy) and from the Terme di Comano spa (Italy). As previously shown, the skin microbial community is biogeographically specific for each body site.^4–7^ In our study design we sampled only two well-defined skin sites to reduce variability due to geographical location in contrast to previous psoriatic microbiome surveys that looked for a skin microbiome signature of disease from multiple skin locations.^21–23^ The two sites chosen, as they are common sites for psoriatic manifestation, were the olecranon skin area (herein the elbow), and the retroauricular crease (herein the ear). Interestingly, the elbow has never been characterised metagenomically before, other than by us in a preliminary experiment to confirm the feasibility of our shotgun-sequencing approach for this site.^32^ Both left and right samples were taken for each patient at these two sites; to overcome the limitations due the high inter vs. intra-subject variability when looking at skin microbiomes, we enroled only patients with at least one of the four sampled areas not directly affected by psoriatic plaques. We chose this approach as we consider unaffected skin regions of an individual to be a better control than an unrelated healthy individual. A clinical assessment and questionnaire was completed for each patient collecting several parameters, including age (avg 56.1, s.d. 12.9), gender (19 males, 9 females), disease severity (Psoriasis Area Severity Index (PASI) index),^34^ and direct presence or absence of psoriatic lesions at the sample site (a summary of the patients recruited and sampled is given in Supplementary Table S1, and full metadata in Supplementary Table S2). The PASI index for the patients ranged from 2 to 35 with an average of 8.6 (s.d. 6.8). We consider PASI <  10 mild, PASI  ≥ 10  <  20 moderate, and PASI ≥ 20 severe psoriasis.^35^ DNA was extracted for each sample, and where sufficient material was obtained (87% of the collected samples, see “Methods“) the DNA was subjected to Illumina 100 nt paired-end sequencing. We report patient characteristics in Table 1 and an overview of metagenomic sequencing is given in Supplementary Table S3.

**Table 1:** Summary of patient characteristics

	Age range (average ± s.d.)	Age range of onset (average ± s.d.)	PASI range (average ± s.d.)	BSA range (average ± s.d.)	Antibiotic usage (# of patients)	Arthritis (# of patients)
Male (*n* = 19)	32–78 (56.4 ± 13.6)	5–70 (35.6 ± 15.0)	2–35 (8.3 ± 7.6)	2–60 (17.0 ± 15.8)	3	5
Female (*n* = 9)	34–80 (55.6 ± 12.7)	15–60 (36.8 ± 15.6)	4–20 (9.1 ± 5.4)	10–50 (22.2 ± 13.0)	1	2
Total (*n* = 28)	32–80 (56.1 ± 12.9)	5–70 (36.0 ± 14.6)	2–35 (8.6 ± 6.8)	2–60 (18.7 ± 14.9)	4	7

In total, 97 individual metagenomic libraries were sequenced obtaining a total of 2.6 billion reads (average reads per sample 26.9 M s.d. 20.0 M), characterised both functionally and taxonomically and subjected to novel strain-level computational profiling methods (see Methods). Of these, 48 samples were collected from sites with visible lesions, whereas the other 49 samples were not directly affected by psoriatic plaques and are herein referred to as unaffected skin samples. Disease presence was more commonly associated with the elbow than the ear (diseased elbow, *n* = 37; unaffected elbow, *n* = 12; diseased ear, *n* = 11; unaffected ear, *n* = 37) as reported in Supplementary Table S1. A greater proportion of non-human sequenced reads (expressed as a percentage of total number of sequenced reads) were obtained for unaffected skin (23.12 s.d. 27.85%) compared to diseased (4.89 s.d. 10.76%) regions (Welch *t*-test, *p*  < 0.0001) (Supplementary Table S3) probably due to a greater proportion of human skin cells being removed by sampling inflamed skin regions compared to unaffected skin areas.

### The species-level structure and composition of the skin microbiome in psoriasis

**Fig. 1:**
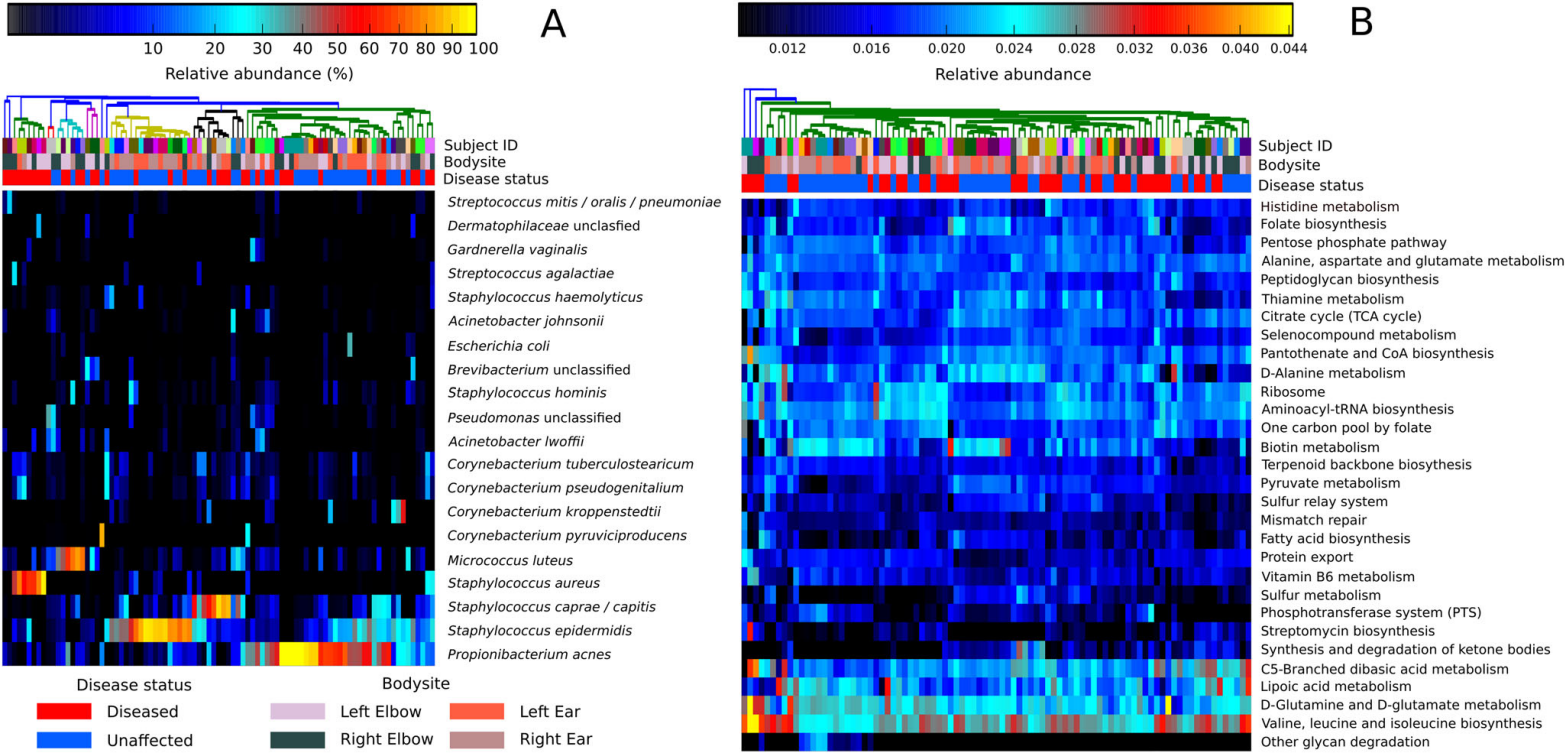
Taxonomic and functional diversity of skin metagenomes. **a** Taxonomic profiles as characterised by MetaPhlAn 2 displaying the 20 most abundant species. Samples are clustered using Bray–Curtis similarity. **b** Functional profiles as characterised by HUMAnN displaying the 30 most abundant KEGG pathways. Samples are clustered based on Euclidean distance

The skin is complex with taxonomically diverse microbial communities. Irrespective of psoriasis present at the site of sampling, we observe by taxonomic analysis (using MetaPhlAn 2,^32, 36^ see Methods) that the cutaneous metagenomes are dominated by the phyla *Actinobacteria* and *Firmicutes*. In particular, the most abundant species are *Staphylococcus epidermidis*, *Propionibacterium acnes*, *S* *taphylococcus* *caprae/capitis*, and *Micrococcus luteus* (Fig. 1a), and eukaryotic species of the fungus genus *Malassezia*. In our study all recruited patients suffer from psoriasis, but the presence and prevalence of *S. epidermidis* and *P. acnes* are in accordance with previous studies profiling cutaneous microbiomes from healthy subjects.^6, 7^

We assessed the overall functional potential of the sampled skin microbiome using the HUMAnN pipeline,^37^ which exploits the richness of shotgun metagenomics to estimate the relative abundances of KEGG pathways^38^ for each metagenome (most abundant pathways are reported in Fig. 1b). As expected, many of the identified KEGG pathways that are common to all samples are highly conserved housekeeping or “core” pathways, including functionalities for sugar metabolism, replication, and translational capabilities. Other more variable, less abundant, and potentially environmentally driven niche-specific functions include antibiotic biosynthesis (e.g., ko00311, penicillin and cephalosporin biosynthesis; ko00521, streptomycin biosynthesis) and heavy metal transport (e.g., M00319, manganese/zinc/iron transport and M00246, nickel transport).

### Microbiome diversity in psoriatic lesions

**Fig. 2:**
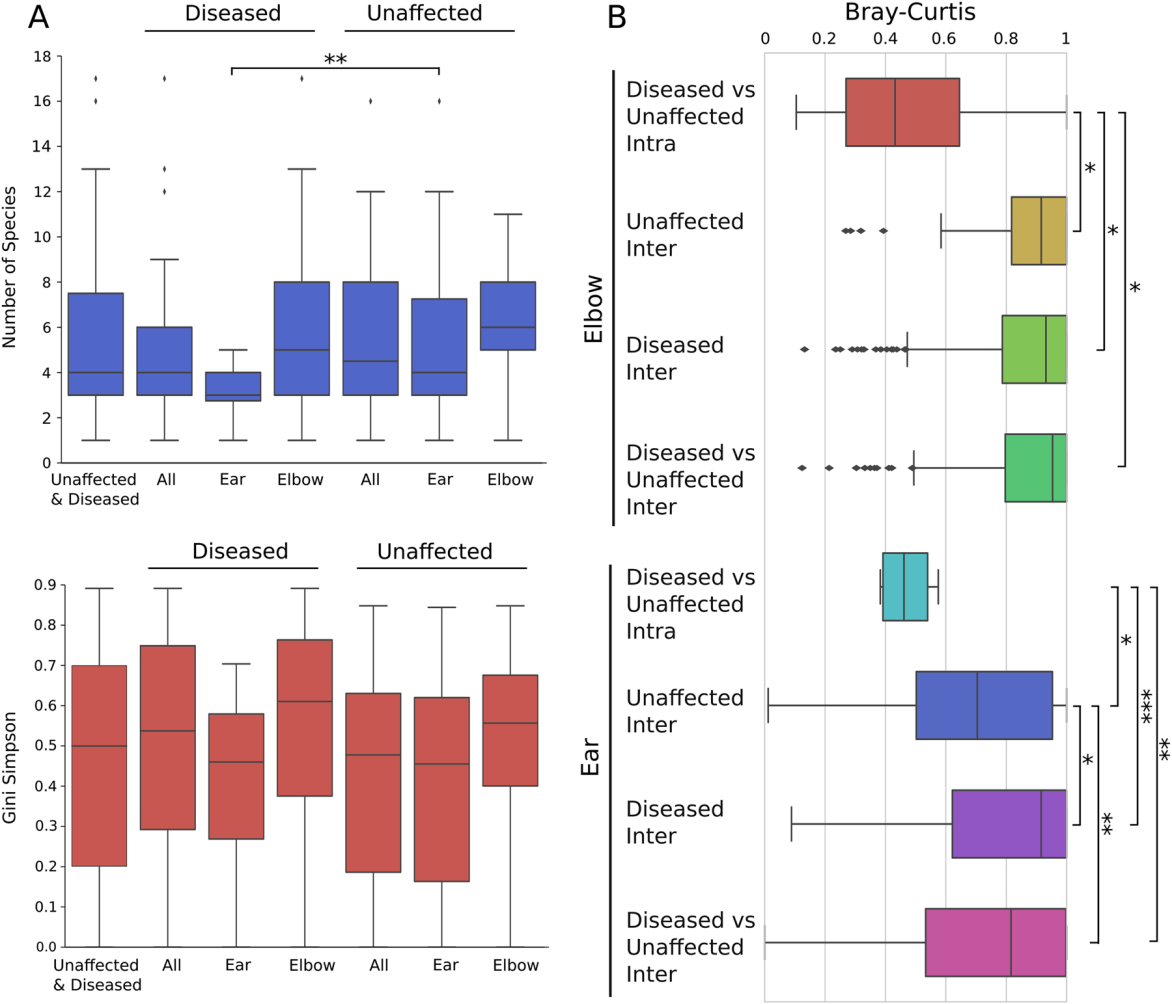
Skin microbial alpha diversity is decreased on ear psoriatic plaques and beta diversity reveals a strong intra-subject specificity. **a** Alpha diversity boxplots of the total number of species (richness) and Gini–Simpson index (evenness) for all samples from the unaffected and diseased ear and elbow skin areas. **b** Beta-diversity boxplots based on Bray–Curtis distances of metagenomics samples from the same patient (intra-beta diversity) and between patients (inter-beta diversity). Levels of significance: *p < 0.05, **p < 0.01, ***p < 0.001

We then investigated whether microbiome diversity was altered in psoriatic lesions. To determine the alpha (within-sample) microbial diversity in relation to disease and body site, we compared the two conditions using both the total number of species (richness) as well as the Gini–Simpson diversity index, which also considers the relative proportion of each species (evenness). At the species level, there is a shift towards decreasing richness in relation to disease status for the ear (*p* = 0.008), but not for the elbow skin areas (Fig. 2a). Notably species richness did not significantly correlate with disease severity (mild, moderate, or severe PASI score) (Supplementary Fig. S1). Importantly, the observed reduction in diversity was not due to low read counts from psoriatic lesions, as diversity indexes were computed on metagenomes subsampled at the same minimum depth 50 K reads (rarefaction was performed after removing human DNA contamination). Although alpha rarefaction curves (richness) indicate that for some samples this level underestimates the microbial richness (Supplementary Fig. S2), principal component analysis highlights that even at 50 K reads the predominant microbial communities can be reproducibly characterised within the metagenomic samples (Supplementary Fig. S3). The higher species richness observed at the previously unsampled elbow compared to the ear (irrespective of whether from psoriatic plaques or unaffected skin) reflects the differences in the microenvironments of the two areas. The ear is a sebaceous area rich in the substance sebum, which lubricates the area as well as provides an antibacterial barrier,^2^ whereas the elbow skin is highly desiccated. This confirms previous findings of decreased microbial diversity at sebaceous sites,^5^ but it is also likely that the elbow harbours more transient species due to environmental exposure and direct environmental contact.

As well as within sample diversity, considerable microbial heterogeneity was observed between the metagenomic samples (Fig. 1a). This is evident comparing the beta diversity distributions of the metagenomes sampled for each patient (intra-subject beta-diversity) and the metagenomes of all distinct patients in the cohort (inter-subject beta-diversity). We observe for the ear, but not the elbow body site, a larger beta diversity in diseased samples compared to unaffected skin (Fig. 2b). Interestingly, when patients presented with disease at only one of the contralateral locations (e.g., one psoriatic and one unaffected elbow), comparison of the distances demonstrated that regardless of disease status at that site the microbial signature of each individual (intra-subject variation) is significantly more similar than comparative sites of other individuals (inter-subject variation) (Fig. 2b). This indicates there is an underlying individuality that influences the microbial composition, as observed in previous studies,^4, 6, 7^ and validates our ad hoc study design, which allows for comparison of the microbiomes from matched unaffected/diseased sites within the same individual.

### Taxonomical and functional microbial changes in psoriatic lesions

The skin microbiome is dominated by members of the genera *Corynebacterium*, *Micrococcus*, *Staphylococcus*, and *Propionibacterium*, the proportions of which vary markedly between individuals (inter-personal variation, Fig. 1a). To counter this variation in our study design, we sampled from both left and right body sites of individuals. Selecting the individuals in the cohort that are inflicted at one contralateral location with respect to the other enabled comparison of the taxonomic differences between psoriatic plaques and unaffected skin (see Methods). We find that members of the genus *Staphylococcus* are significantly more abundant on diseased skin compared to unaffected skin (Wilcoxson signed-rank test *p*  = 0.023 after correcting for multiple hypothesis testing with respect to the other abundant genera). This is also true when only elbow pairings are compared (*p*  = 0.043). While all previous surveys report differences associated with psoriatic lesions and healthy controls,^21–25^ all concur with this study that there is no specific strong biomarker associated with disease. In fact, at the species-level resolution we find no significant differences between psoriatic and unaffected skin.

To explore the community-wide functional differences associated with psoriatic lesions, we applied LEfSe,^39^ a biomarker discovery tool (see Methods). We observed a number of significant functional differences at both the ear and elbow in relation to disease. At the ear, KEGG pathways involved primarily in biodegradation and metabolism were increased compared to unaffected skin, including Benzoate (ko00362), Naphthalene (ko00626), and Lysine degradation (ko00310). In unaffected ears metabolism of vitamins and cofactors was more prevalent, e.g., Riboflavin (B2) (ko00740), and, as might be expected, healthy ears are lipid-rich and have lipid metabolism (ko00600). Such differences in broad functionality likely reflect nutrient availability on diseased and unaffected skin. At the elbow, where there is a higher species richness compared to the ear, we found the community functioning between diseased and unaffected metagenomes to be more uniform; although there are differences in functionalities including carbohydrate and amino acid metabolism, intriguingly, we identify that bacterial secretion (ko0370) and protein export (ko03060) are more prevalent in unaffected compared to diseased skin microbiomes.

**Fig. 3:**
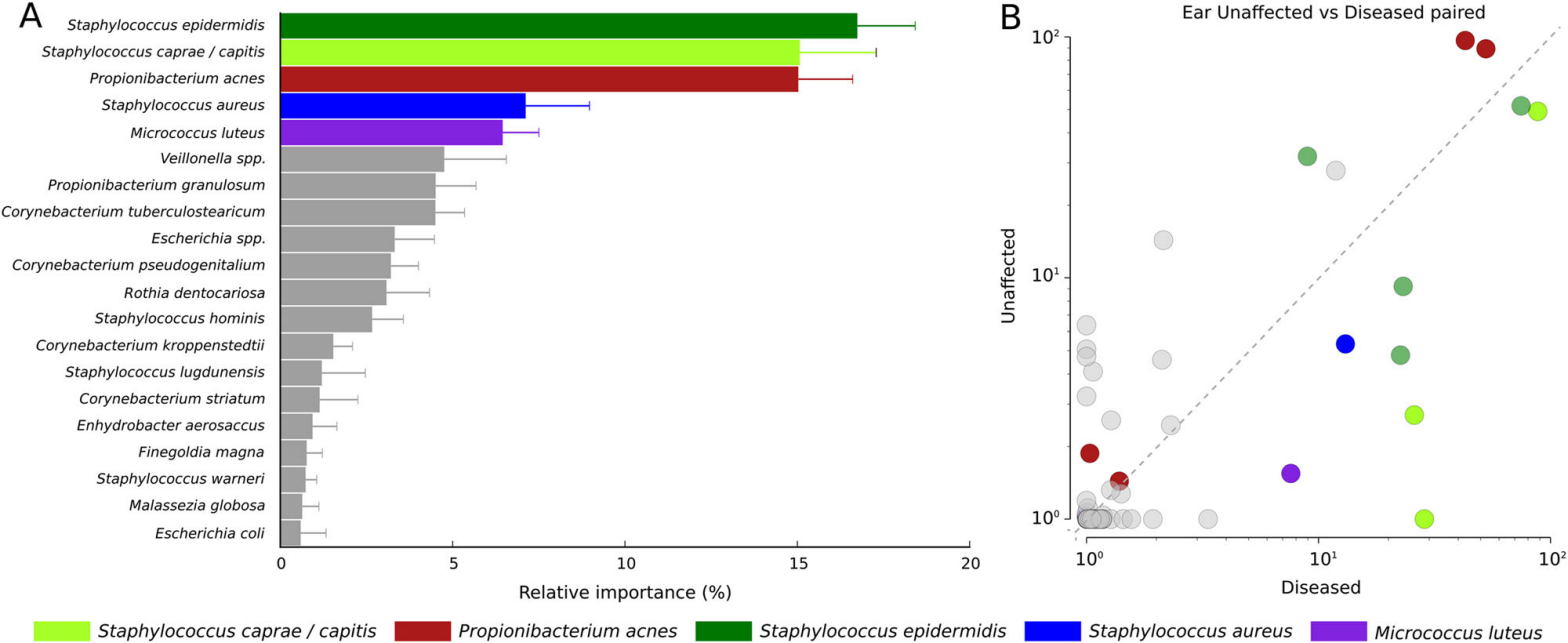
Taxonomic discrimination of unaffected and diseased ears. **a** Top five taxonomic discriminatory features (species) as determined by Random Forest analysis and the relative importance of each feature. **b** Scatterplot comparing the relative abundances of each shared species between patients presenting with one unaffected and one diseased ear. *Coloured circles* refer to the top five most discriminatory species

To further investigate any microbial signatures associated with psoriasis-affected regions, we employed a classification and feature selection approach based on Random Forests (RF)^40, 41^ (see Methods) to identify both the key taxonomic and functional variables discriminating the samples with respect to disease (Supplementary Fig. S4A). In classifying all diseased against all unaffected samples, the Random Forests classifier gave a better-than-random discrimination for diseased vs. unaffected skin (area under the ROC curve (AUC): 0.630). However, when separating the samples on body site there was no predictive power for classifying diseased or unaffected samples from the elbow, but there was for the ear (AUC: 0.619). The most discriminatory taxonomic features as determined by RF for separating diseased and unaffected ears were *S. caprae/capitis*, *P. acnes*, *S. epidermidis*, *S. aureus*, and *M. luteus* (Fig. 3a) and the association of these species to diseased or unaffected ears is shown in Fig. 3b. Therefore, while there is limited evidence of overall qualitative changes in either the taxonomic or functional composition of the skin microbial communities at least for the ear body site, there is an indication that psoriasis does have an effect on quantitative changes at the species level in the most dominant skin commensals. However, it is unclear if these changes are a direct result of disease (i.e., host–microbe interaction) or as a consequence of a shift in the skin’s abiotic conditions.

In addition to disease status, there are many patient characteristics that can influence an individual’s microbial signature, including disease severity, age, body site, gender, and general health. As the skin is biogeographical,^4–7^ as expected, Random Forest gave a high predictive accuracy for separation based on body site for taxonomic features (AUC: 0.744) and functional features (KEGG modules, AUC: 0.811) (Supplementary Fig. S4B), we did not, however, find any discriminatory power with respect to gender or disease severity, body surface area (BSA) or PASI (mild, moderate, or severe).

### Strain-level variations of psoriatic and unaffected skin locations within the same patient

**Fig. 4:**
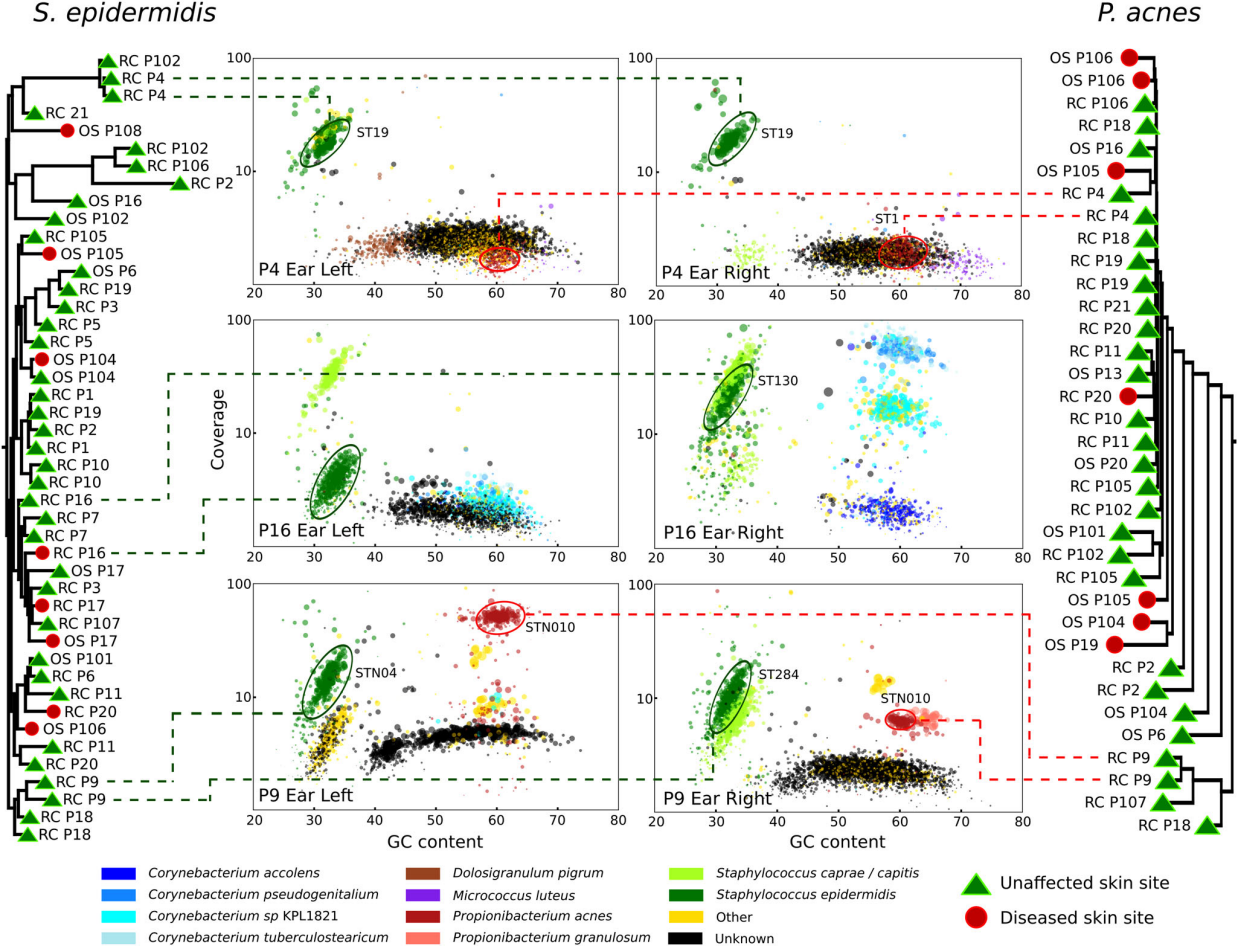
Strain-level diversity of *P. acnes* and *S. epidermidis*. Assembly-based and marker-based analyses of paired ear samples from three patients highlights strain conservation in *right* and *left* sides of subjects with psoriatic unaffected ears (Patients 4 and 9), but suggests diversification in relation to disease (patient 16, one psoriatic and one unaffected ear). The internal scatterplot panels report all contigs assembled for each sample, coloured according to assigned taxonomic species. The contigs are ordered by GC content and the average coverage in the metagenomic samples, *black* contigs indicate unknown taxonomy. Using species-specific markers, phylogenetic trees for *P. acnes* and *S. epidermidis* were built from the metagenomes, where the species are present and at sufficient depth of coverage to permit analysis. Similarly, if coverage depth permitted, MLST types (ST numbers) were assigned. RC and OS refers to ear and elbow metagenomics samples, respectively, and patient number is prefixed with P

The presence of only a few highly dominant species that are common to both unaffected and psoriatic skin may suggest that crucial differences are present at the strain level only. It is known that typical intra-species genomic variability can result in wide range of phenotypic diversity, including virulence, substrate utilisation, antibiotic production, or susceptibility.^42–44^ Although shotgun metagenomics can greatly improve the taxonomic resolution afforded by ribosomal-based PCR surveys,^26^ metagenomic strain-level profiling is still an open computational challenge.^45^ Here, to explore intra-species distribution within and between individuals we applied a set of strain-level profiling methods we developed.^46–48^ The first recently developed approach is an assembly free strain-level phylogenetic method based on species-specific MetaPhlAn 2 markers^32, 36^ called StrainPhlAn,^46^ which identifies strains looking at taxon-specific single-nucleotide polymorphisms (SNPs) (see Methods) and allowed us to build two whole-genome phylogenetic trees of the two most common skin species *S. epidermidis* and *P. acnes* (Fig. 4) from the samples with sufficient coverage (roughly > 2x). To complement the phylogenetic information, we implemented an assembly-based reconstruction method with mapping-based taxa post-processing (see Methods), and an assembly-free metagenomic multi-locus sequence typing (MLST) profiler, MetaMLST.^47^

These tools allowed us to explore the strain-level features of the skin microbiome. This can be first illustrated by applying them on the three paired samples with the highest sequencing depths (Fig. 4). First, we observe that the unaffected left and right ears of patients P4 and P9 are colonised by the same strain of *S. epidermidis*. P9 is also colonised by the same strain of *P. acnes*, whereas for P4 it is more difficult to obtain conclusive identities due to the low abundance of *P. acnes* in this patient (4.8 and 6.7% in the left and right samples, respectively). For patient 16 who presented with one diseased and one unaffected ear, we notice a different *S. epidermidis* strain colonising each site. The MLST profiles we retrieved were in agreement with the phylogenetic placement in all instances except for patient 9, where MLST analysis differentiated the types colonising the left and right ear, most likely due to presence of the closely related species *S. caprae*/*capitis* in one of the samples. Different strains colonising diseased and unaffected skin within an individual is supportive of the idea of possible niche-specific strain-level selection in psoriatic disease. Strain heterogeneity may not always occur in all species in all cases: for instance, the diseased and unaffected elbow pairs of patients 104 are colonised by the same strain of *S. epidermidis* but, perhaps relevantly, different strains of the more abundant *P. acnes* (Fig. 4). Subspecies diversity is an intriguing observation (not available and therefore missed with ribosomal community profiling), and, although anecdotal, it is clear there exists strain-level heterogeneity of the skin and this may be relevant to disease. While a larger cohort size is required to fully explore this hypothesis, our results nonetheless highlight the importance of strain-level analyses to uncover the true diversity of the skin microbiome. Moreover, strain-level profiling enables the investigation of the specific functional capacity of strains inhabiting psoriatic and unaffected skin, as further analysed in the next section.

### Functional variability of *S. epidermidis* strains colonising psoriatic and unaffected skin

Different strains are likely characterised by different functional repertoires, and to further investigate the specific genes associated with strain-level niche selection, we applied our recently developed and validated tool, PanPhlAn^48, 49^ (see Methods) that characterises the functional potential of specific microbial strains within a metagenomic sample. It provides the set of genes that are present or absent for the strain of interest based on the pangenome for that species. We focused on the investigation of the functional potential of *S. epidermidis* strains from diseased and unaffected skin, as this abundant skin species displayed the highest strain heterogeneity (Fig. 4). In total, 128 single-gene differences were identified (Fisher exact test, *p* < 0.05), 50 of which were more prevalent in strains inhabiting psoriatic plaques (Supplementary Fig. S5) compared to 78 from strains found on unaffected skin (Supplementary Fig. S6).

At psoriatic sites we found *S. epidermidis* strains containing known virulence-related genes. For instance, two strains occupying diseased skin, but absent from all strains found at unaffected sites, harbour a putative *Staphylococcus* secretory antigen gene, *ssaA*. SsaA is an extracellular protein that has shown to be implicated in pathogenesis.^50^ Similarly, two strains from diseased sites have putative *esxA* genes. EsxA is a virulence factor in *S. aureus* that has also been identified and shown to be functional in some *S. epidermidis* strains.^51^ Generally, we observe multiple markers of horizontal gene transfer (HGT) in strains from psoriatic skin, including transposases, integrases, and other phage-related genes. In line with HGT events, we also identify genes frequently associated with mobile genetic elements, including multi-drug resistance transporters (MFS DHA2 multi-drug resistance, lantibiotic ABC transporter), penicillin-binding proteins, and a beta-lactamase. Importantly, this is not a consequence of patient treatment, as patients receiving antibiotic treatments were excluded from this analysis.

We also noted a number of putative transcriptional regulators and genes of unknown function that, while uncharacterised, could nonetheless be of environmental relevance. Indeed, it is not uncommon for genes partitioned in the horizontal gene pool and presumed environmentally selected to be poorly characterised. It is evident that in combination with *S. epidermidis* strain heterogeneity colonising the skin of psoriatic patients, there is also considerable functional diversity. It is plausible to consider the differences in functional capacity between strains associated with unaffected skin and psoriatic plaques to be the consequence of niche adaptation or selection.

### Psoriatic microbial niches comprise a large proportion of unknown microbes

**Fig. 5:**
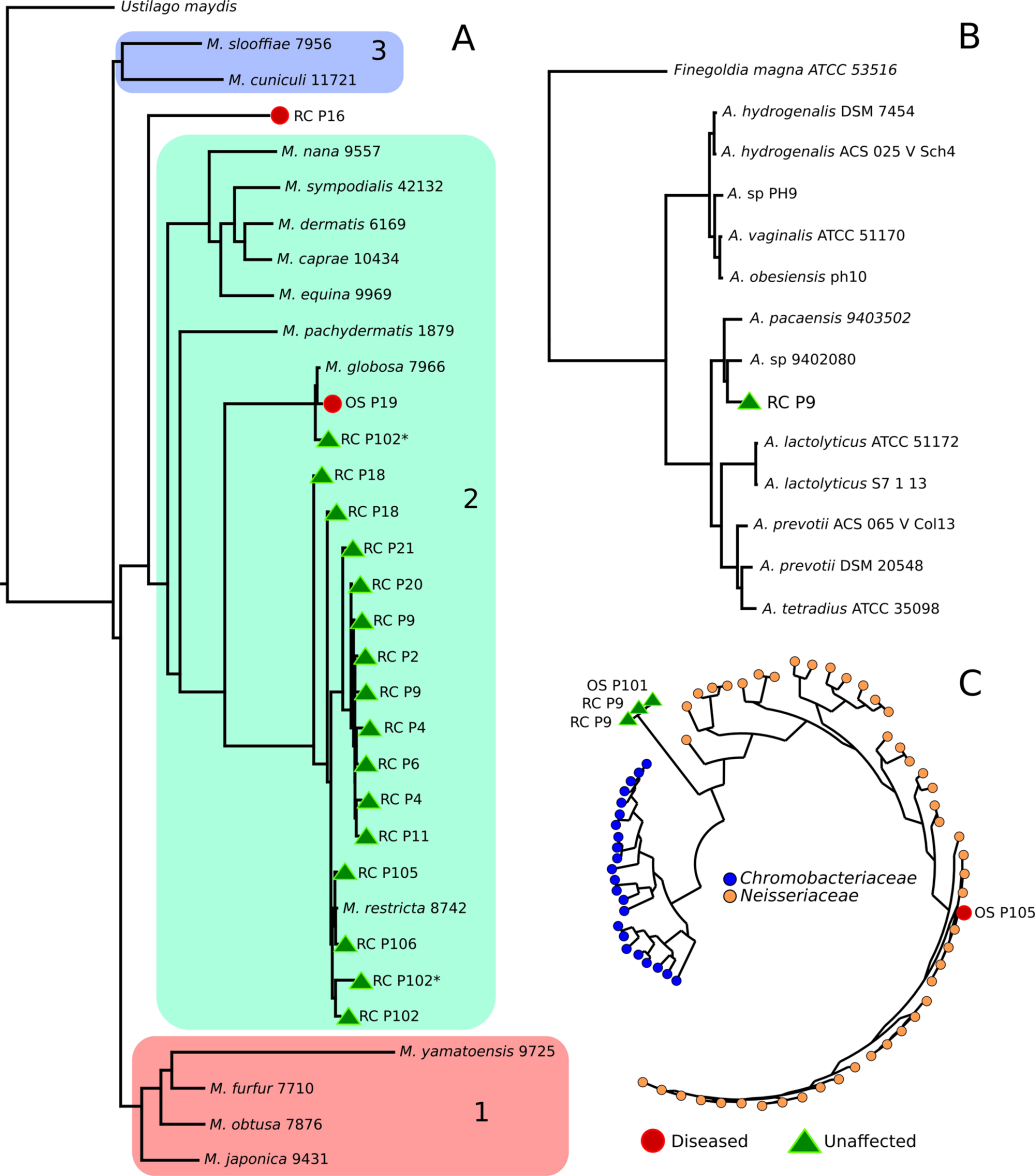
Phylogenetic analysis of taxonomically uncharacterised metagenomic assemblies (unknowns) compared to the closest representative reference genomes. **a** Phylogenetic tree of “unknown” eukaryotic assemblies compared to reference *Malassezia* genomes. The inclusion of the *Malassezia* spp. and *Ustilago maydis* available reference genomes in the tree shows that unaffected and diseased skin is colonised by uncharacterised *Malassezia* and *Malassezia* spp. Marked with *asterisk* indicate the two *Malassezia* genomes reconstructed from the patient’s 102 left ear. *Malassezia* clusters, 1–3, are congruent with those reported previously,^54^ with most of the *Malassezia* reconstructed genomes falling within cluster 2. **b** Phylogenetic tree of “unknown” bacterial assemblies in the *Peptostreptococcaceae* family. *Anaerococcus* spp. and *Finegoldia magna* reveal a novel *Anaerococcus* spp. on the ear of patient 9. **c** Phylogenetic tree of members of the *Chromobacteriaceae* (23 species) and *Neisseriaceae* (47 species) and “unknown” assemblies from Patients 9 and 101 are unable to be placed in either family

The skin is inhabited by diverse taxa and intra-species variability that is poorly characterised. This “dark matter” includes species, genera, or higher-level taxonomic ranks with either no or only a few representative references genomes. By employing an assembly-based genome reconstruction approach for each skin metagenome (see Methods), we identified contig clusters with little or no homology to the reference data sets (Fig. 4), which, therefore, represent taxa without any closely related sequenced strains. To explore these “unknowns” further, we first compared the assemblies to the closest available references based on sensitive mapping capturing even at low sequence similarities. This enabled us to identify a number of uncharacterised but abundant eukaryotic and bacterial organisms from the cutaneous microbiomes. A common, eukaryotic inhabitant of the skin microbial community is the fungus *Malassezia globosa*.^52^ Where sufficient coverage permitted, we identified 18 “unknown” clusters with either weak or divergent genome content compared to *M. globosa*. Their reconstructed genomes averaged in length 4.4 Mb (s.d. 2.8 Mb), which means a large fraction of the genome was reconstructed given the draft genomes obtained from pure culture of *M. globosa* CBS 7966^53^ and *M. restricta* CBS 8742 are 8.96 and 7.26-Mb long, respectively.^54^ Recently, representative genomes for all 14 accepted species of the *Malassezia* genus have been sequenced,^54^ whereby the authors report phylogenetically the *Malassezia* genus supports three main clusters. Phylogenetic comparison of our *Malassezia* reconstructed genomes (Fig. 5a) finds most fit in cluster 2 and are closest to *M. globosa* and *M. restricta*, the two most common *Malassezia* spp. found on human skin.^54^

Unexplored diversity is still, however, present within the metagenomically retrieved *Malassezia* genomes in cluster 2 (Fig. 5a). Only four of the reconstructed genomes are placed close enough (Average Nucleotide Identity (ANI)^55^ > 97.5%) to *M. globosa* (patient 19 and one strain from patient 102) or *M. restricta* (Patients 105 and 106) to be confidently assigned to these two species. For the other *Malassezia* genomes, e.g., the highest ANI of the strains from Patient 9 compared to the most phylogenetically related *M. restricta* 8742 is 92.84% (s.d. 2.52%), which is suggestive that they may represent a distinct and unsequenced species. More strikingly, patient 16’s ear is instead inhabited by a more distantly related strain, which cannot be assigned to any of the three clusters, and therefore could be an unknown more ancestral *Malassezia* spp. (Fig. 5a); intriguingly, this ear was diseased and may therefore be colonised by a hitherto uncharacterised fungal species, which may be of relevance to psoriatic disease.

Focusing on unknown bacterial clusters, we detected a cluster in Patient 9 that likely represents an uncharacterised *Anaerococcus* spp. (Fig. 5b) as its closest reference is *Anaerococcus* spp. 9402080, but the two genomes only share an ANI value of 80.6% (s.d. 5.28%). Both ears of Patient 9 are also inhabited by an unknown bacterial taxon that is related but cannot be placed in either the *Chromobacteriaceae* or *Neisseriaceae* families (Fig. 5c). What emerges from the analysis of the “microbial dark matter” is that the skin microbiome, both unaffected and in relation to disease, is much more complex and diverse than taxonomic profiling based on references genomes alone permits. Thus, such hidden diversity highlights limitations in our reference data sets and the potential role of these unknown taxa in skin health and disease could be overlooked.

## Conclusions

It is becoming increasingly evident that the resident microbiome, and dysbiosis thereof, is an important aspect in disease such as inflammatory bowel diseases,^28^ obesity,^29^ and type 2 diabetes.^27^ While considerable efforts have been made to assess the role of the gut microbiome in relation to disease, the skin microbiome with respect to human health and disease is still largely unexplored. In part this is due to difficulties in recovering sufficient material from skin samples and contaminating human cells that have hindered metagenomic studies. Thus, most knowledge of the cutaneous microbiome has been gained through culture-dependent methods or low-resolution marker gene approaches. However, seminal work of the Human Microbiome Project,^6^ Oh et al.^7^ and recently ourselves^32^ have demonstrated the cutaneous microbiome can be analysed in depth. Yet despite the potential of shotgun metagenomics it has not been applied to the study of the microbiome associated with psoriasis.

For the first time we have applied community-wide whole-genome metagenomics to taxonomically and functionally characterise the microbiome associated with plaque psoriasis. Consistent with previous studies,^6, 7, 32^ the skin harbours a taxonomically and functionally diverse community of micro-organisms and we identify that psoriatic plaques at the ear are characterised by a significant decrease in microbial diversity. Although the causality remains unclear, a lowering of diversity with respect to disease is common, being observed in many other disease-associated microbiomes, including the gut of individuals suffering Crohn’s disease,^56^ necrotising enterocolitis,^57^ and those suffering from psoriatic arthritis.^58^

We identified quantitative changes in association with psoriasis, particularly an increase in *Staphylococcus* and predictive power to discriminate psoriatic and unaffected ears. However, at the species level no specific biomarker indicative of disease was identified. Indeed, irrespective of disease status we identify a stronger underlying subject-specific microbial signature defining the microbiome. It must, however, be noted in this study that unaffected and diseased samples were taken from psoriatic patients, and as psoriasis is a systemic condition the microbial signatures of plaques compared to healthy individuals free of the disease may be more marked. Using finer and novel strain-level techniques we highlighted strain heterogeneity within individuals and between psoriatic and unaffected skin. We demonstrated for the common and abundant species *S. epidermidis* that this strain diversity is accompanied by potentially environmentally relevant functional differences that may be indicative of niche-specific selection with respect to psoriatic lesions.

As for many microbiome studies the diversity of reference genomes available from the public databases for comparison can only capture a small fraction of the in vivo biodiversity, which is highlighted in this study. With ad hoc developed pipelines for genome reconstruction and analysis, we have identified a number of uncharacterised eukaryotic and bacterial taxa. These include a potentially new *Anaerococcus* spp. and a bacterial taxon related to but unable to be grouped in either the *Chromobacteriacea* *e* or *Neisseriaceae* families, as well as potentially novel *Malassezia* spp. and a distant taxon related to *Malassezia* from a psoriatic lesion raising the possibility it could be niche specific. Our study suggests that more efforts should be devoted on targeting those community members that are poorly represented in the public databases, including the uncultivated members of the skin microbiome by single-cell sequencing approaches^59^ or by developments in cultivation methods, as these currently uncharacterised organisms might be relevant in disease pathogenesis.

This study provides a first step in utilising shotgun metagenomics to unravel the involvement of the skin microbiome in psoriasis. It highlights the complexity and diversity in association with the disease that has hitherto been overlooked using traditional profiling methods at lower resolution. With an increased cohort and depth of sequencing, as well as additional methods to access strain level and the considerable uncharacterised microbial “dark matter”, future metagenomics studies can refine the map of the microbes across populations and disease, with the aim of discovering biomarkers as targets for therapeutic treatments and potential commensal microorganisms that could be utilised for topical probiotics.

## Materials and methods

### Patient recruitment

This study was performed under approval of the Ethical Committee of the University of Trento and all participants gave their informed consent. Male and female patients ranging in age from 32 to 80 were recruited from the Province of Trentino (Italy) by the Santa Chiara Hospital in Trento and by the Terme di Comano. Sampling was performed by the same clinically accredited dermatologist in order to minimise collection biases. Patients provided a medical history and underwent clinical assessment, where the severity of plaque psoriasis was classed according to PASI.^34^ The BSA involved in psoriasis and whether the left and right olecranon skin area (elbow) and retroauricular crease (behind the ear) were free of plaque lesions was also recorded. As psoriasis is a chronic condition, inevitably, patients have varied treatment histories. Supplementary Table S4 indicates if patients were currently undergoing or had received systemic treatment for psoriasis, or used topical creams in the past month, if not patients had neither used systemic or topical psoriasis treatments in at least the last 6 months prior to sampling. In all cases patients were asked to refrain from using any topical treatment in the week preceeding sampling. In addition, four individuals had also undergone a course of antibiotics (for psoriasis or an unrelated condition) in the last 3 months. For the purpose of alpha and beta diversity analysis and biomarker discovery and Random Forest machine-learning analyses (see below), metagenomes from patients who had received antibiotic treatments were excluded. Our rational of comparing diseased and unaffected skin from the same individual was decided to mitigate differences in current or past treatment regimes.

### Sample collection, DNA extraction, and illumina shotgun sequencing

For each patient, samples were taken from the left and right olecranon skin area and retroauricular crease by clinically accredited dermatologists, using a sampling protocol based on the one validated and adopted by the Human Microbiome Project (HMP) consortium.^6^ Sterile cotton tip swabs (VWR, Milan, Italy) were moistened in SCF-1 buffer (50 mM Tris-HCl, pH 7.5; 1 mM EDTA, pH 8.0; 0.5% Tween-20)^6^ and swabbed over the sample site applying pressure for approximately 30 s. To recover the sample the swab head was pressed and rotated against the side of 15-ml sterile collection tube. Samples were initially treated in a lysis solution (20 mM Tris-HCl, pH 8.0; 2 mM EDTA; 1% Triton X-100) containing a final concentration of 20 mg/ml Lysozyme (Sigma-Aldrich, Milan, Italy) and incubated for 30 min at 37 °C. DNA was isolated from each sample using the MoBio PowerSoil DNA isolation kit (MoBio Laboratories, Carlsband, CA, USA) as previously described.^6^ Laboratory control extractions were also performed on prepared sample buffer and swabs to ascertain any potential contaminants. Each metagenome was first quantified and where there was sufficient material (> 1 ng) libraries were prepared using the Nextera-XT DNA kit (Illumina Inc., San Diego, CA, USA) using the manufacturers protocol. Libraries were sequenced (2x 100 bp) on the Illumina HiSeq-2000 platform. Of the 112 possible samples in this study, sufficient material and successful libraries were produced for 97.

### Sequence, sample QC, and subsampling

The generated raw metagenomes were processed with FastqMcf^60^ by trimming positions with quality <15, removing low-quality reads (mean quality <25), and discarding reads shorter than 90 nt. Human and Bacteriophage phiX174 (Illumina spike-ins) DNA were then removed by using BowTie2^61^ to map the reads against the reference genomes. All metagenomes have been deposited and are available at the NCBI Sequence Read Archive under accession ID SRP057859 (BioProject PRJNA281366).

### Taxonomic and functional profiling

Metagenomic samples were taxonomically profiled using MetaPhlAn 2^32, 36^ with default parameter settings. MetaPhlAn is a tool that maps shotgun reads to a database of unique clade-specific markers and is capable of producing species-level resolution and the relative abundances of each species/clade in a sample. The profiling results are reported in Fig. 1a (top 20 organisms) and are based on the fraction of reads mapping against the taxonomically unique species-specific MetaPhlAn2 markers (average 1.7 s.d. 0.84%) (Supplementary Table S5) consistently with previous reports.^32, 36^ To functionally profile the metagenomes we used HUMAnN^37^ (with default parameter settings), which is based on KEGG pathways and modules.^38^ Initially reads were mapped against the KEGG database using USEARCH 7^62^ (31.5 s.d. 14.0% matching reads) (Supplementary Table S5) before HUMAnN collapsed the output producing a table of functions and their relative abundances in each metagenome.

### Diversity analysis

To ensure fairness of comparison alpha and beta diversity was calculated on samples rarefied to 50 k reads (after human DNA removal) each, reducing the data set from 97 to 89 metagenomes, as eight samples had total read numbers <50 k. In the case of patient 14 there were no samples where the depth coverage was ≥ 50k reads, thus this reduced the number in the participants from 28 to 27. Total species richness for each sample was calculated as the sum number of species identified using MetaPhlAn 2 (using conditions above) and *p*-values presented are from the Welch’s *t*-test. Species evenness was calculated using the Gini–Simpson index. To produce alpha diversity rarefaction curves, the data set was rarefied at incremental levels and the alpha diversity (species richness) calculated. Diversity between metagenomes from the same individual (intra-beta diversity) and between individuals (inter-beta diversity) were computed using Bray–Curtis distance matrix.^63^

### Biomarker and machine-learning analysis

#### Biomarker discovery

LEfSe (LDA effect size),^39^ a metagenomics biomarker discovery tool was used with default parameters to discover the functional genomic features associated with unaffected and diseased skin at the elbow and ear.

Due to strong individual-specific microbial signatures, for taxonomic comparison between diseased and unaffected sites, taxonomic differences were compared between patients presenting with a diseased and a corresponding unaffected contralateral site. To insure the most accurate taxonomic assessment possible, the paired samples were rarefied to the lowest sequencing depth of the pairing before taxonomic characterisation using MetaPhlAn 2,^32, 36^ e.g., patient 16’s diseased ear metagenome comprises 2.8 million non-human reads, and the unaffected ear metagenome 8.14 million non-human reads, therefore the later metagenome was randomly subsampled to 2.8 million reads. Differences in taxonomic abundances were calculated using the Wilcoxson signed-rank test for the abundant skin genera *Propionibacterium*, *Staphylococcus*, and *Micrococcus,* and species therein *P. acnes*, *S. epidermidis*, *S. aureus*, *S. caprae/capitis*, and *M. luteus*.

#### Random forest classification

We evaluated the prediction strength of metagenomic data in linking the skin microbiome with disease state or skin location. We investigated this task through a Random Forest (RF)^40^ machine-learning classification approach in which we used species abundance or functional features to discriminate between unaffected and diseased samples.^41^ Due to the sensitivity of this analysis to the sample sequencing depth, this was performed on the 50 K rarefied data set (samples were rarefied to 50 K after removal of human reads). The free parameters of this classifier were set as follows: (i) the number of decision trees was equal to 500; (ii) the number of features to consider when looking for the best split was equal to the root of the number of original features; (iii) the quality of a decision tree split was measured using the gini impurity criterion. Feature importance was computed using the “mean decrease impurity” strategy.^64^ These importance values were considered to perform an embedded feature selection strategy (denoted as RF-FS) implemented as follows: (i) RF was applied on the entire set of features; (ii) features were ranked in terms of importance; (iii) RF was trained again on the top *k*-th features by varying them in the set (5, 10, 20, 30, 40, 50, 60, 70, 80, 90, 100, 125, 150, 175, 200); (iv) the number of features that maximised the accuracy was chosen as the optimal number; (v) the new model was generated by training RF on this reduced set.

The entire analysis was done through a cross-validation approach. In particular, prediction accuracies were assessed by 10-fold cross-validation, repeated and averaged on 20 independent runs. Classification performances were evaluated in terms of AUC statistic, which can be interpreted as the probability that the classifier ranks a randomly chosen positive sample higher than a randomly chosen negative one, assuming that positive ranks higher than negative.

The software framework used for these experiments has been described elsewhere,^41^ is open-source and available online at http://segatalab.cibio.unitn.it/tools/metaml.

### Strain-level profiling

#### Assembly-based reconstruction with post-processing taxa mapping

Each metagenomic sample was processed with de-novo SPAdes genome assembler (version 3.1.1; using default parameters)^65^ which compared to other assemblers gave the best results in our extensive tests on synthetic and real metagenomes (data not shown). Taxonomy was assigned to each contig by a procedure, which was developed with conservative choices to minimise false positive assignments. More specifically, we applied BLASTN^66^ to map the assembled contigs in each sample against all the microbial reference genomes (including bacteria, archaea, viruses, and microeukaryotes) available in the NCBI repository as of August 2015. The mapping results were post-processed to compute the breadth of coverage (fraction of the query contig covered) and nucleotide sequence identity for each contig with respect to the best matching (draft) genome in the considered reference database. We assign a species-level taxonomic label “A” to a contig if it matches at least one of the available reference genomes for species “A” with breadth of coverage and percentage identity both higher than 90%. For the three most abundant bacterial species (*S. epidermidis*, *S. aureus*, *P. acnes*), we binned the contigs based on their taxonomic labels as for these species a large number of reference genomes are available and it is thus highly unlikely that long contigs (>1000 nt) do not match any reference genome in the sample. For these species, we also curated the set of reference genomes with PhyloPhlAn^67^ to exclude wrongly labelled genomes. Manual inspection further validated each bin for consistency on the contig GC-content vs. coverage plots and based on the overall length of the contig in the bin. Preliminary binning of contigs without reliable taxonomic labels was performed based on the GC content and coverage of each contigs using a *K*-means clustering algorithm. We manually curated this set of preliminary bins by selecting and further analysing only the best bins that showed high GC content and coverage consistency (less than 5% deviation on the GC content and 25% on the coverage) and discrete separation from other bins. We analyse the phylogenetic placement (Figs. 4, 5) of only these manually curated contig bins. Details for the assembled genomes, from each metagenomes, for the five most prevalent skin inhabitants (*S. caprae/capitis*, *P. acnes*, *S. epidermidis*, *S. aureus,* and *M. luteus*) are given in Supplementary Table S5.

#### MLST analysis of metagenomic data

We performed an assembly-free multi-locus sequence typing analysis on the metagenomic samples using MetaMLST^47^ by mapping the reads against the publicly available MLST house-keeping genes sequences of *P. acnes* from PubMLST (*aroE*;* atpD*;* gmk*;* guaA*;* lepA*;* sodA*;* tly*;* CAMP2*, 162 sequences)^68–70^ and *S. epidermidis* (*arcC*;* aroE*;* gtr*;* mutS*;* pyr*;* tpi*;* yqiL*, 330 sequences).^71^ The mapping was performed with the local alignment mode of Bowtie2, version 2.1.0 (options: -D 20 -R 3 -N 0 -L 20 -i S,1,0.50).^61^ From the alignment we were able to obtain a consensus sequence for every MLST locus in each sample. The sequence was built following a majority rule for the positions covered by more than one read, while the closest reference sequence was used as template for positions with zero coverage. The sequences of each locus were then profiled together to define a Sequence Type (ST) for each sample, taking into account duplicated and recurring (even potentially new) sequences. Strains were considered identical if more than six of eight MLST loci were identical and any loci did not differ by more than one SNP.

#### Functionally profiling of S. epidermidis skin strains

PanPhlAn^48^ is a computational tool to reconstruct strain-specific gene repertoires from metagenomic samples with an assembly-free pangenome-based approach. In brief, PanPhlAn estimates the abundance of each pangene family associated with at least one strain of a given species and uses the co-abundance principle to define the set of pangenes specific of the strains present in the sample. The PanPhlAn profiles for *S. epidermidis* were generated specifying the following set of parameters: --min_coverage 1, --left_max 1.70, and --right_min 0.30. Strain-specific genes more statistically significantly associated with *S. epidermidis* strains inhabiting diseased or unaffected skin were determined by Fisher exact test (*p*  < 0.05). To ascribe function, each gene sequence was mapped against the KEGG database^38^ using USEARCH 7^62^ and NCBI non-redundant database with BlastX, and subsequently manually annotated.

#### P. acnes and S. epidermidis phylogenetic trees

The *P. acnes* and *S. epidermidis* phylogenetic trees were built by applying RAxML^72^ (with parameters “–m GTRCAT –p 1234”) on the sample-specific variants of species-specific marker genes extracted from the samples and the reference genomes performed using StrainPhlAn.^46^ For each metagenome, the reads were first mapped against the MetaPhlAn 2 markers by Bowtie2^61^ and the mapping post-processed^73^ to produce the consensus sequence for the mapped reads. These consensus sequences represent the most abundant strains present in a sample for each species. Similarly, the MetaPhlAn 2 markers were mapped against the reference genomes^66^ to obtain the strain-specific variants of the marker genes for each strain previously sequenced from pure culture. Finally, the strain-specific markers reconstructed from genomes and metagenomes were aligned by MUSCLE^74^ and concatenated to form the species strains.

### *Malassezia* spp. phylogenetic tree

Groups of taxonomically unassigned contigs from assembly-based reconstruction (see above) whose best (although weak) hit was to *Malassezia globosa* CBS 7966^53^ were further analysed. The 18 groups of unknown *Malassezia* spp. identified with sufficient sequence coverage were compared to *Malassezia* reference genomes (*M. globosa* CBS 7966^53^ and 13 other accepted *Malassezia* spp.^54^) and the related *Ustilago maydis* 521.^75^

The phylogeny was generated using the *M. globosa* CBS 7966^53^ as a reference to compute the core genome. Specifically, *M. globosa* CBS 7966 genes were aligned to all analysed genomes using BLASTN (evalue < 1e−05, word size 9). Sequences longer than 500 bp and with an identity higher than 70% were considered as plausible orthologous genes. Orthologous genes present in more than 95% of the analysed genomes were considered as part of the core genome and were extracted, concatenated, and aligned using MUSCLE.^74^ The resulting multiple alignment was trimmed using trimAl^76^ with the option “-gappyout” to remove unwanted gaps. The phylogenetic tree was built using a maximum-likelihood approach implemented in RAxML.^72^ RAxML was executed using the parameter “-m GTRGAMMA” and 50 bootstrap steps.

### *Anaerococcus* and *Neisseriacea*/*Chromobacteriaceae* phylogenetic trees

To estimate the phylogenetic placement of the new assemblies, each assembly with an average coverage >3X was processed by a newly extended version PhyloPhlAn.^67^ This version is specifically designed to take as input both genomes and proteomes (i.e., the whole set of translated gene sequences from the genome) and map them against its internal reference database of 400 universal proteins. In this instance the “unknown” bacterial assemblies were placed in the *Anaerococcus* and *Neisseriaceae*/*Chromobacteriacea* phylogenetic trees. Both phylogenetic trees were built using FastTree (parameters: -quiet -bionj -slownni -mlacc 2 -spr 4).^77^ The *Neisseriaceae*/*Chromobacteriaceae* phylogenetic tree was visualised using GraPhlAn.^78^

## Electronic supplementary material

Supplementary Table S2

Supplementary Material

Supplementary Table S5
