# Supplementary material for: Unexplored diversity and strain-level structure of the skin microbiome associated with psoriasis
Source: NPJ Biofilms Microbiomes. 2017 Jun 22;3:14. doi: 10.1038/s41522-017-0022-5 (PMC5481418; doi:10.1038/s41522-017-0022-5)
Supplement: Supplementary file 2 — Supplementary Material [file 41522_2017_22_MOESM2_ESM.docx]

Unexplored diversity and strain-level structure of the skin microbiome associated with psoriasis

Adrian Tett ^1^, Edoardo Pasolli ^1,^^, Stefania Farina ^2,^^, Duy Tin Truong ^1,^^, Francesco Asnicar ^1^**,** Moreno Zolfo ^1^, Francesco Beghini ^1^, Federica Armanini ^1^, Olivier Jousson ^1^, Veronica De Sanctis ^3^, Roberto Bertorelli ^3^, Giampiero Girolomoni ^4^, Mario Cristofolini ^2^, Nicola Segata ^1,*^

*1 Centre for Integrative Biology, University of Trento, Italy*

*2 Istituto G.B. Mattei, Comano, Italy*

*3 NGS Facility, Laboratory of Biomolecular Sequence and Structure Analysis for Health, Centre for Integrative Biology, University of Trento, Italy*

*4 Department of Medicine, Section of Dermatology, University of Verona, Italy*

*^ These authors contributed equally*

** Corresponding author* [*nicola.segata@unitn.it*](mailto:nicola.segata@unitn.it)

**Supplementary Figures and Tables**

**Supplementary Tables**

|  | **Samples collected** | | **Samples above 50K reads** | |
| --- | --- | --- | --- | --- |
| **Patient sample(s) collected and successfully sequenced** | **Number of patients** | **number of metagenomes** | **Number of patients** | **number of metagenomes** |
| One diseased elbow sample | 4 | 4 | 3 | 3 |
| Two diseased elbow samples | 12 | 24 | 10 | 20 |
| One unaffected elbow sample | 3 | 3 | 4 | 4 |
| Two unaffected elbow samples | 0 | 0 | 0 | 0 |
| One diseased and one unaffected elbow sample | 9 | 18 | 7 | 14 |
|  | **28** | **49** | **24** | **41** |
|  |  |  |  |  |
| One diseased ear sample | 2 | 2 | 2 | 2 |
| Two diseased ear samples | 2 | 4 | 2 | 4 |
| One unaffected ear sample | 2 | 2 | 2 | 2 |
| Two unaffected ear samples | 15 | 30 | 15 | 30 |
| One diseased and one unaffected ear sample | 5 | 10 | 5 | 10 |
|  | **26** | **48** | **26** | **48** |

**Supplementary Table S1. Summary of the recruited patients and their disease status at each body-site.** Number of patients refers only to samples for which a successful shotgun metagenome was obtained (total 28 patients and 97 metagenomes), number of patients for which the metagenomics sequencing depth exceeded 50k reads is also given (total 27 patients and 89 metagenomes). In total at least one successful elbow metagenome was produced from each of the 28 patients, for the ear at least one metagenome was produced from 26 patients.

**Supplementary Table S2 (See attached Excel file).** Full sample/patient metadata

|  | **Sample number (n)** | | **Reads (k) after QC (Average ± S.D)** | | **Read % as proportion of total (Average ± S.D)** | |
| --- | --- | --- | --- | --- | --- | --- |
|  | **All** | **>50K** | **All** | **>50K** | **All** | **>50K** |
| Disease | 48 | 41 | 26,765 (± 21,414) | 28,713 (± 22,578) | 4.89 (± 10.76) | 5.68 (± 11.47) |
| Ear | 11 | 11 | 23,863 (± 19,970) | 23,863 (± 19,970) | 5.52 (± 9.16) | 5.52 (± 9.16) |
| Elbow | 37 | 30 | 27,628 (± 22,012) | 30,491 (± 23,524) | 4.70 (± 11.30) | 5.75 (± 12.35) |
|  |  |  |  |  |  |  |
| Unaffected | 49 | 48 | 27,081 (± 18,752) | 27,322 (± 18,873) | 23.12 (± 27.85) | 23.60 (± 27.95) |
| Ear | 37 | 37 | 27,237 (± 19,428) | 27,237 (± 19,428) | 26.08 (± 29.81) | 26.08 (± 29.81) |
| Elbow | 12 | 11 | 26,602 (± 17,284) | 27,610 (± 17,754) | 14.00 (± 18.86) | 15.24 (± 19.26) |
|  |  |  |  |  |  |  |
| Total | 97 | 89 | 26,924.7 (± 20,008.3) | 27,963 (± 20,553) | 14.10 (± 22.99) | 15.35 (± 23.61) |

**Supplementary Table S3. Metagenomic Sequencing overview**

| Patient ID | Topical Psoriasis treatment | Systemic Psoriasis treatment | Antibiotic usage |
| --- | --- | --- | --- |
| 1 | 1 | 0 | 0 |
| 2 | 1 | 0 | 0 |
| 3 | 1 | 0 | 1 |
| 4 | 0 | 0 | 0 |
| 5 | 1 | 0 | 0 |
| 6 | 0 | 0 | 0 |
| 7 | 0 | 0 | 0 |
| 8 | 0 | 0 | 0 |
| 9 | 1 | 0 | 1 |
| 10 | 0 | 0 | 0 |
| 11 | 0 | 1 | 0 |
| 13 | 0 | 1 | 0 |
| 14 | 0 | 1 | 0 |
| 15 | 1 | 0 | 1 |
| 16 | 1 | 1 | 0 |
| 17 | 0 | 1 | 0 |
| 18 | 0 | 1 | 0 |
| 19 | 0 | 1 | 0 |
| 20 | 0 | 1 | 0 |
| 21 | 0 | 1 | 0 |
| 22 | 0 | 1 | 0 |
| 101 | 0 | 0 | 0 |
| 102 | 1 | 0 | 0 |
| 104 | 0 | 0 | 0 |
| 105 | 0 | 0 | 0 |
| 106 | 0 | 0 | 1 |
| 107 | 0 | 0 | 0 |
| 108 | 0 | 0 | 0 |

**Supplementary Table S4. Patient treatment history**

**Supplementary Table S5 (See attached Excel file).** Assembly statistics and HUMAnN, MetaPhlAn2 mapping percentages

**Supplementary Figures**

**Supplementary figure S1.** Alpha diversity (species richness) compared to disease severity of the patient (PASI) indicating diversity is not associated with disease severity. All samples are rarefied to 50K sequences.

**
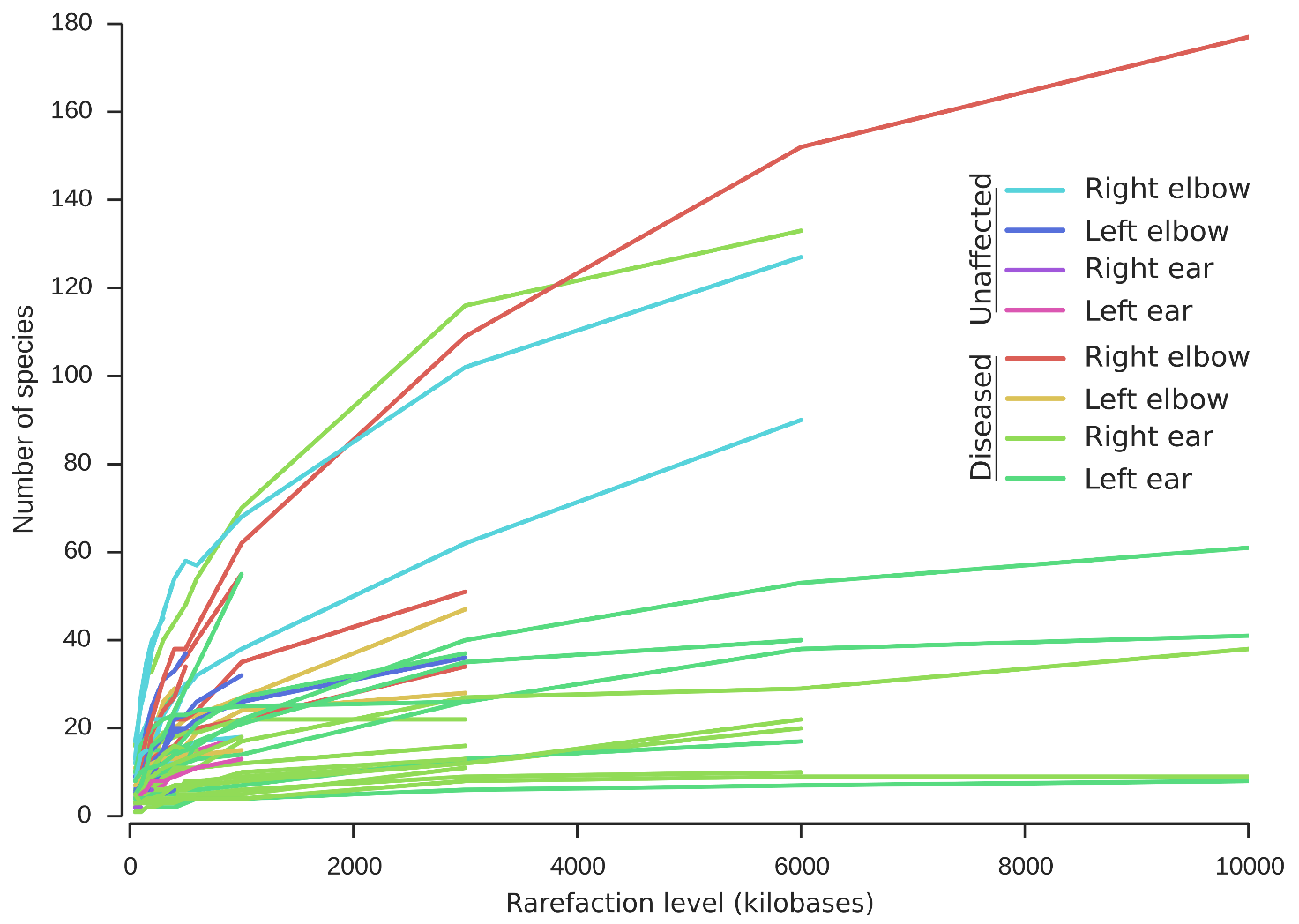
**

**Supplementary Figure S2.** Alpha diversity rarefaction curves of diseased and unaffected ear and elbow samples.

**
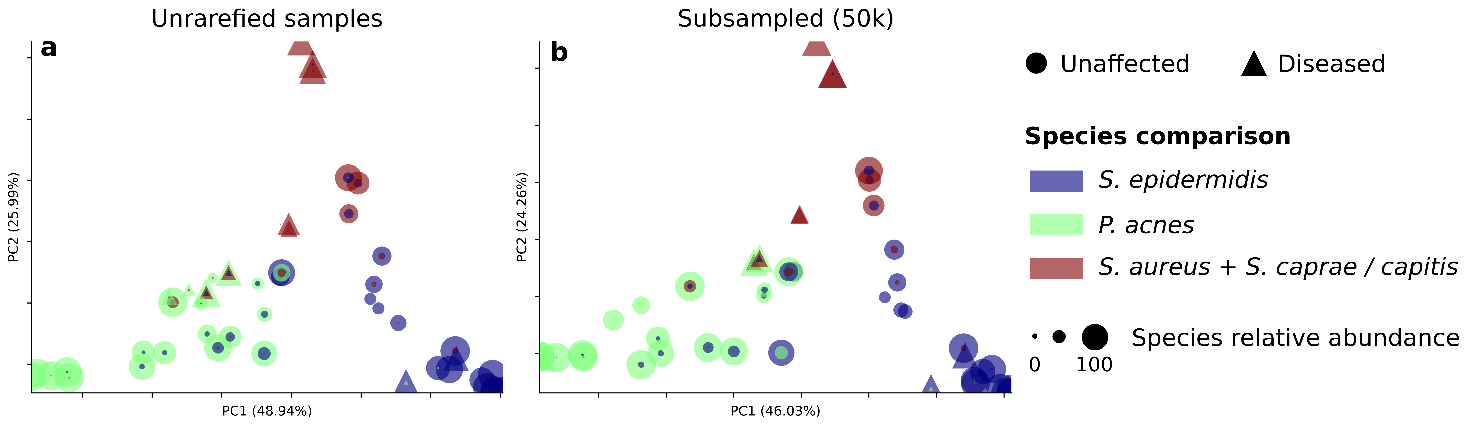
**

**Supplementary Figure S3**. Principal component analysis (PCA) based on taxonomic profiles of the metagenomics samples in this study. Panels **A** and **B** Samples are coloured according to the relative proportion of *Staphylococcus epidermidis*, *Propionibacterium acnes* and *Staphylococcus aureus*, *caprae* and *capitis*, unrarefied or rarefied to 50k respectively, clearly showing that the relative abundance of these organism is largely responsible for the separation of samples. Importantly, even at a 50k rarefaction level (the level selected for alpha and beta diversity comparisons) MetaPhlAn 2^1,2^ can accurately identify the predominant microbial communities within the skin samples of this study, confirming the low impact of rarefaction on assembly-free community composition profiling.^1,2^.


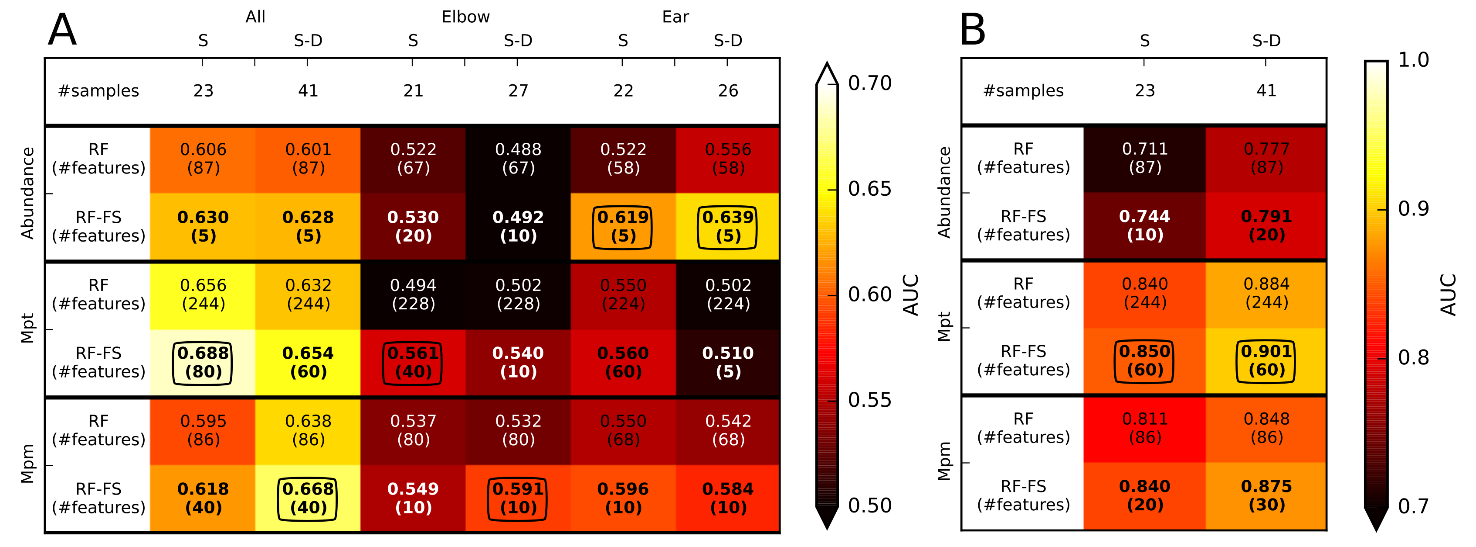


**Supplementary Figure S4. Assessment of the prediction capability of skin microbiome profiles in determining whether a sample has been collected from an unaffected or psoriatic skin site (A) or body site (B).** The random forest classifier ^3^ has been applied and its performances evaluated with the area under the roc curve (AUC) score for all samples simultaneously (“All”) and separately for elbow and ear samples. The “S” columns denote the performances obtained considering in the training data only one sample per patient, whereas for the “S-D” columns two samples per patient (one from a unaffected and one from a psoriatic site) are considered at each cross validation run. The classifier is evaluated using all features (“RF”) and using feature selection in the training phase (“RF-FS”). In parenthesis, we report the total number of features available for RF, and the total number of selected features for RF-FS. Three set of features were considered: taxonomic features as inferred by MetaPhlAn 2 analysis (“Abundance”), pathway abundance ("Mpt") and functional module abundance ("Mpm") as inferred by HUMAnN ^4^ analysis.


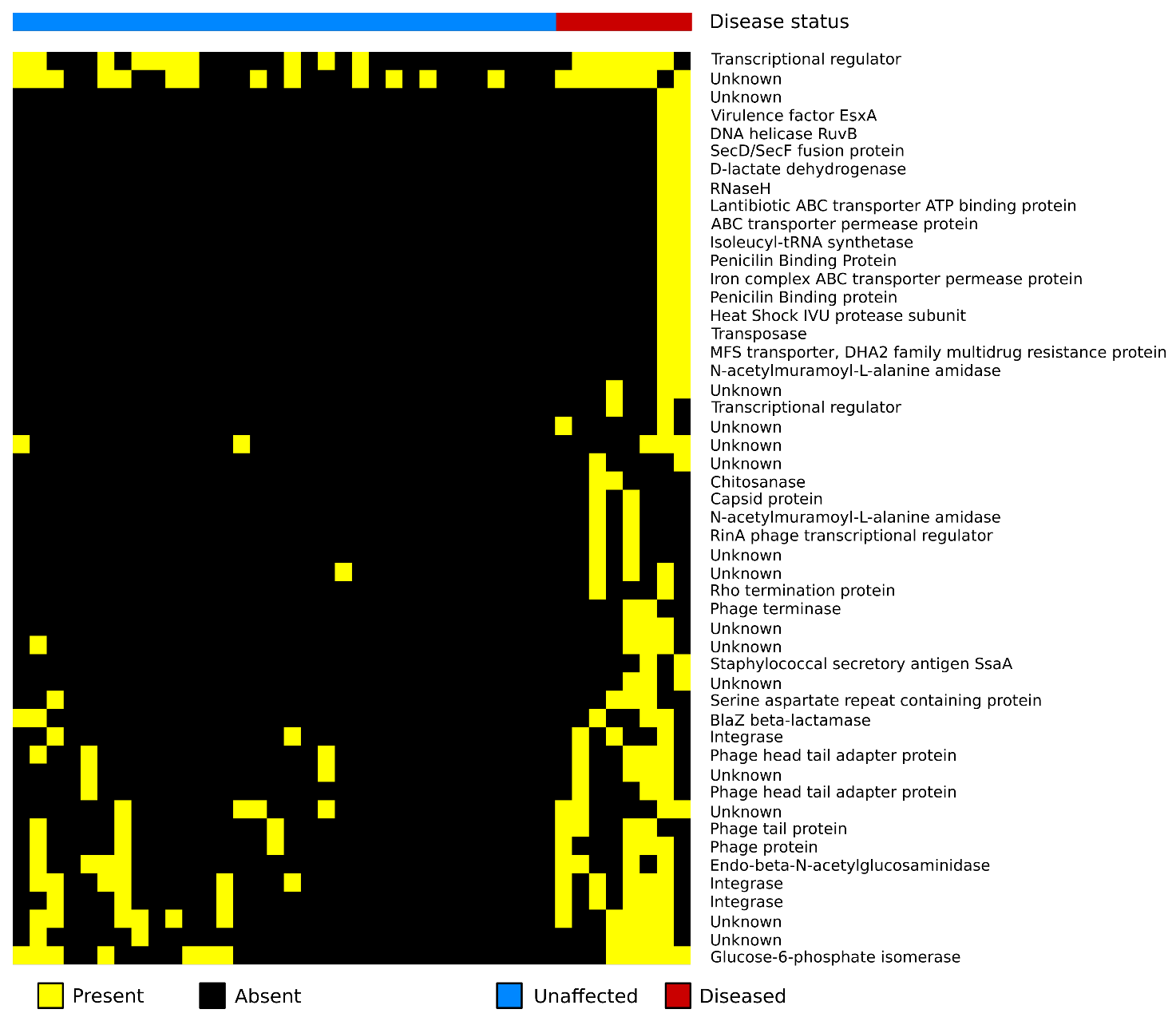


**Supplementary Figure S5**. Functional variability of *S. epidermidis* strains as identified by PanPhlAn^5^. Genes significantly more prevalent (Fisher exact test, p < 0.05) in *S. epidermidis* strains inhabiting psoriatic plaques


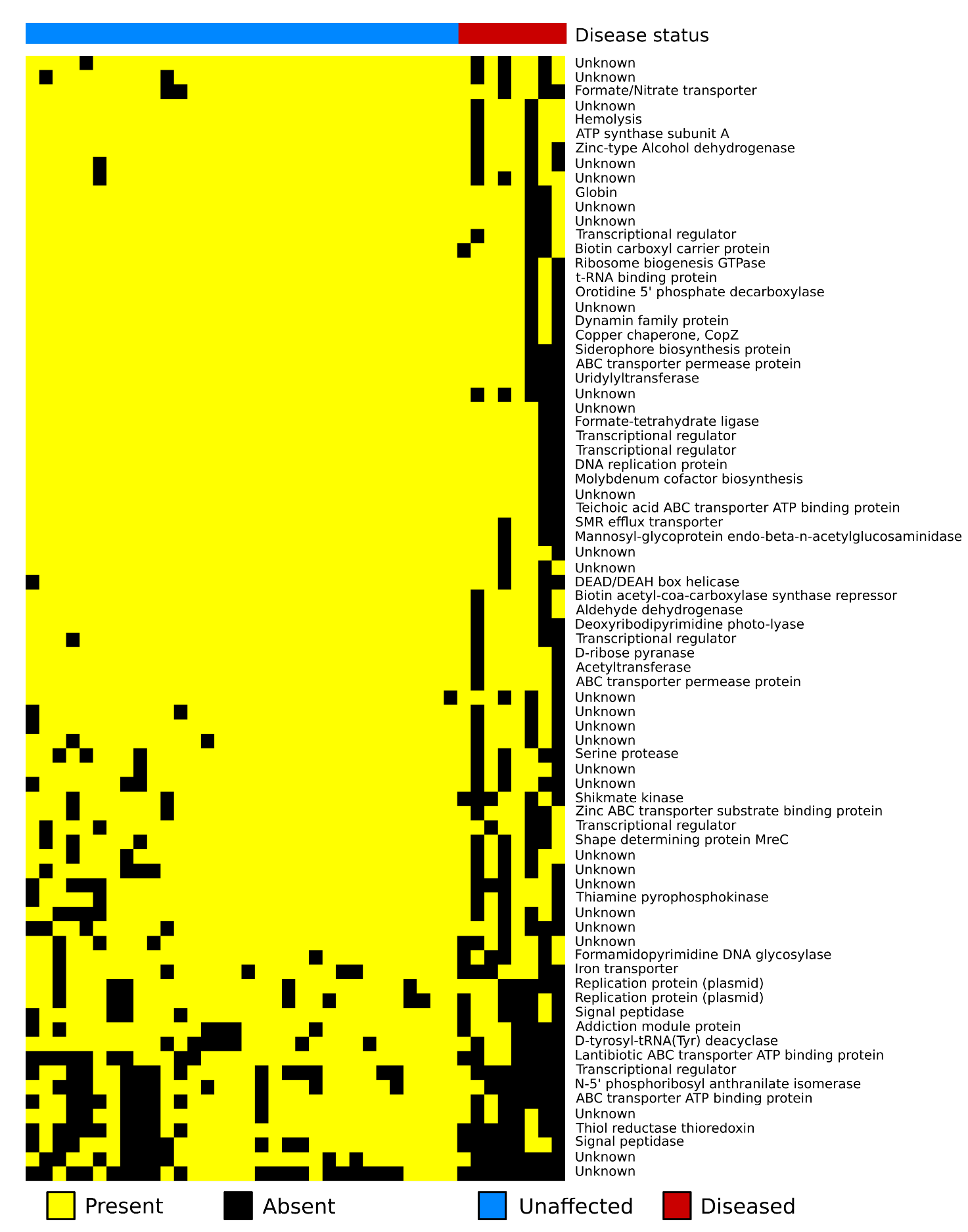


**Supplementary Figure S6**. Functional variability of *S. epidermidis* strains as identified by PanPhlAn^5^. Genes significantly more prevalent (Fisher exact test, p < 0.05) in *S. epidermidis* strains inhabiting unaffected skin.

**Supplementary References**

1 Segata, N. *et al.* Metagenomic microbial community profiling using unique clade-specific marker genes. *Nat Methods* **9**, 811-814, doi:10.1038/nmeth.2066 (2012).

2 Truong, D. T. *et al.* MetaPhlAn2 for enhanced metagenomic taxonomic profiling. *Nat Methods* **12**, 902-903, doi:10.1038/nmeth.3589 (2015).

3 Breiman, L. Random forests. *Mach Learn* **45**, 5-32, doi:Doi 10.1023/A:1010933404324 (2001).

4 Abubucker, S. *et al.* Metabolic reconstruction for metagenomic data and its application to the human microbiome. *PLoS Comput Biol* **8**, e1002358, doi:10.1371/journal.pcbi.1002358 (2012).

5 Scholz, M. *et al.* Strain-level microbial epidemiology and population genomics from shotgun metagenomics. *Nat Methods*, doi:10.1038/nmeth.3802 (2016).
